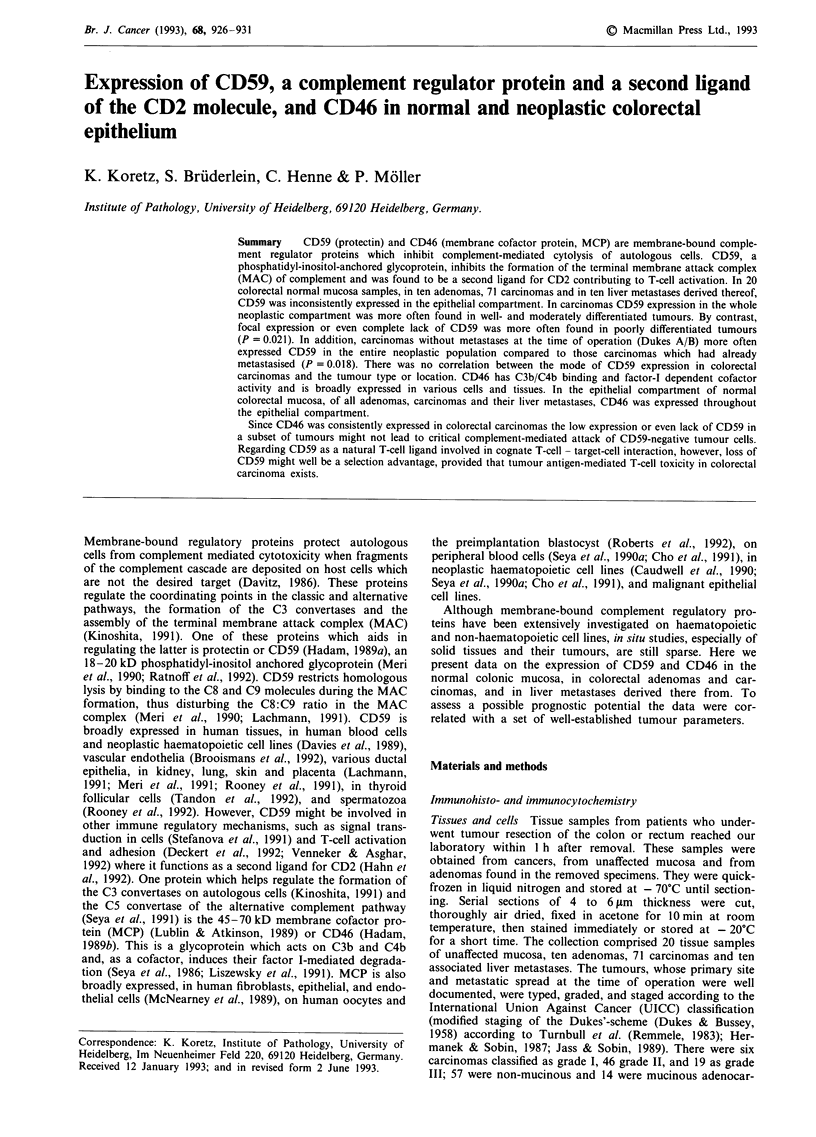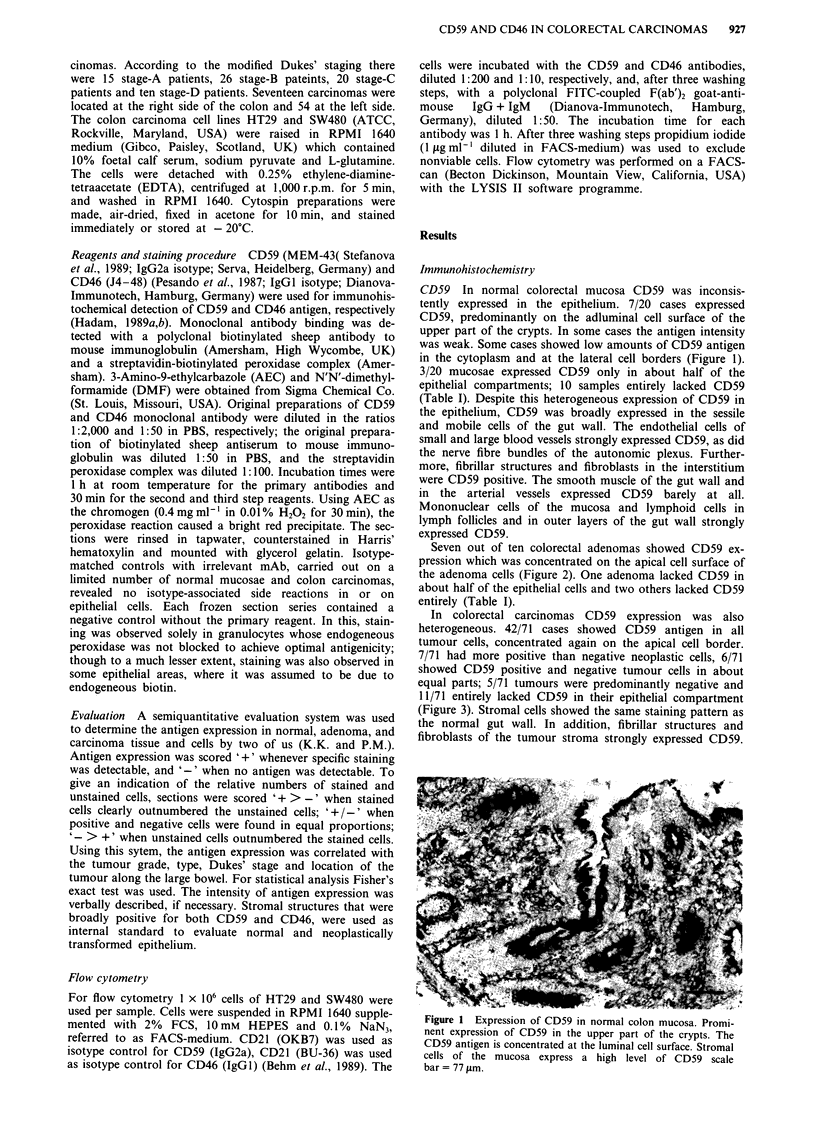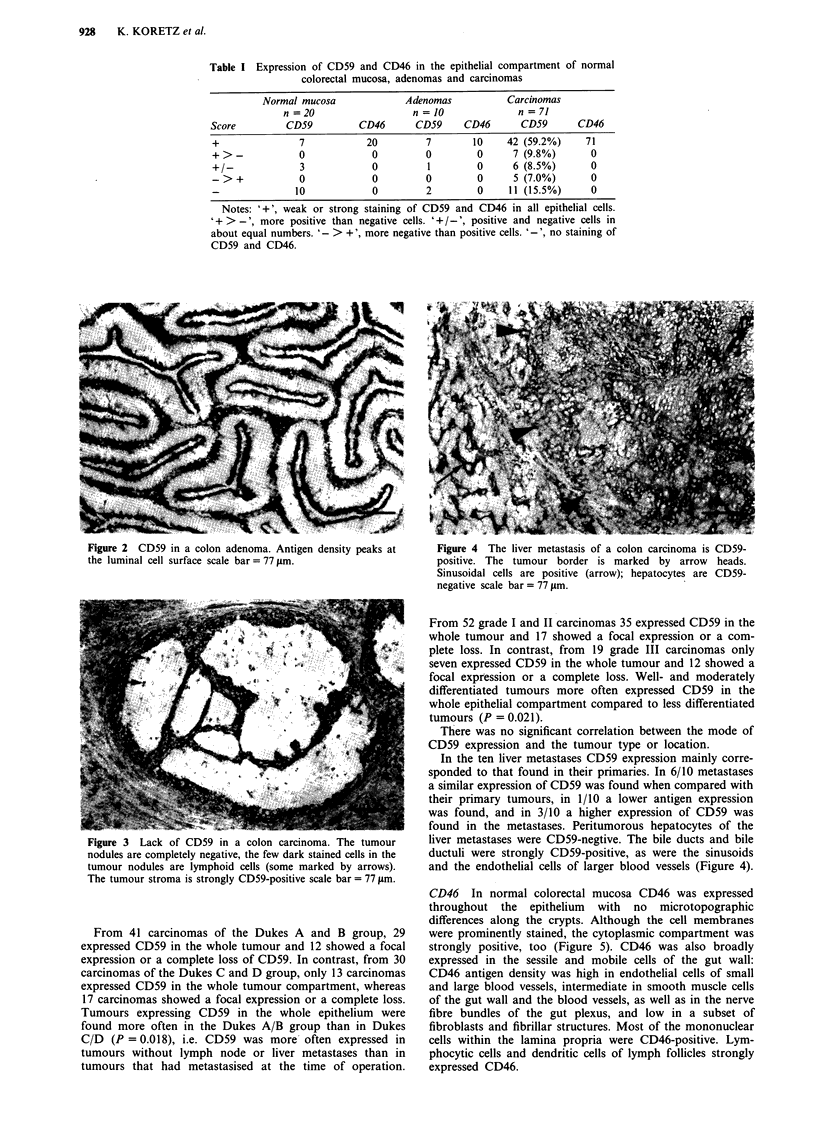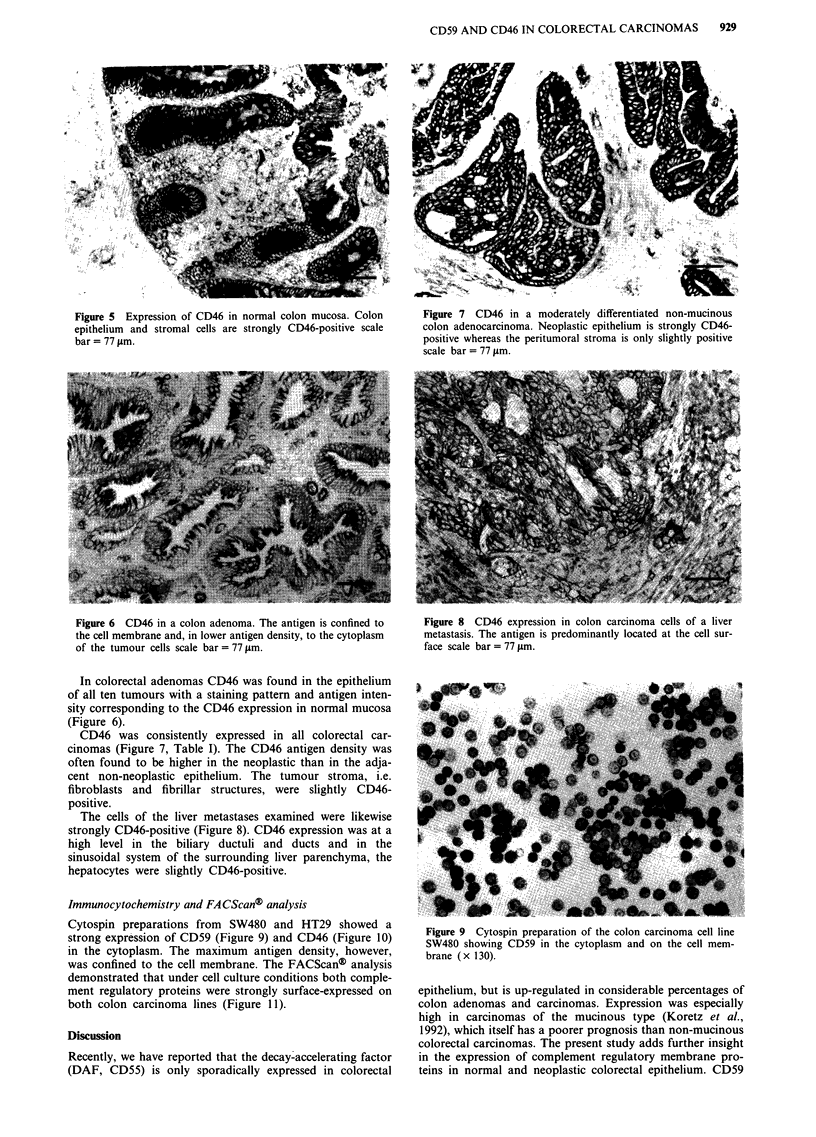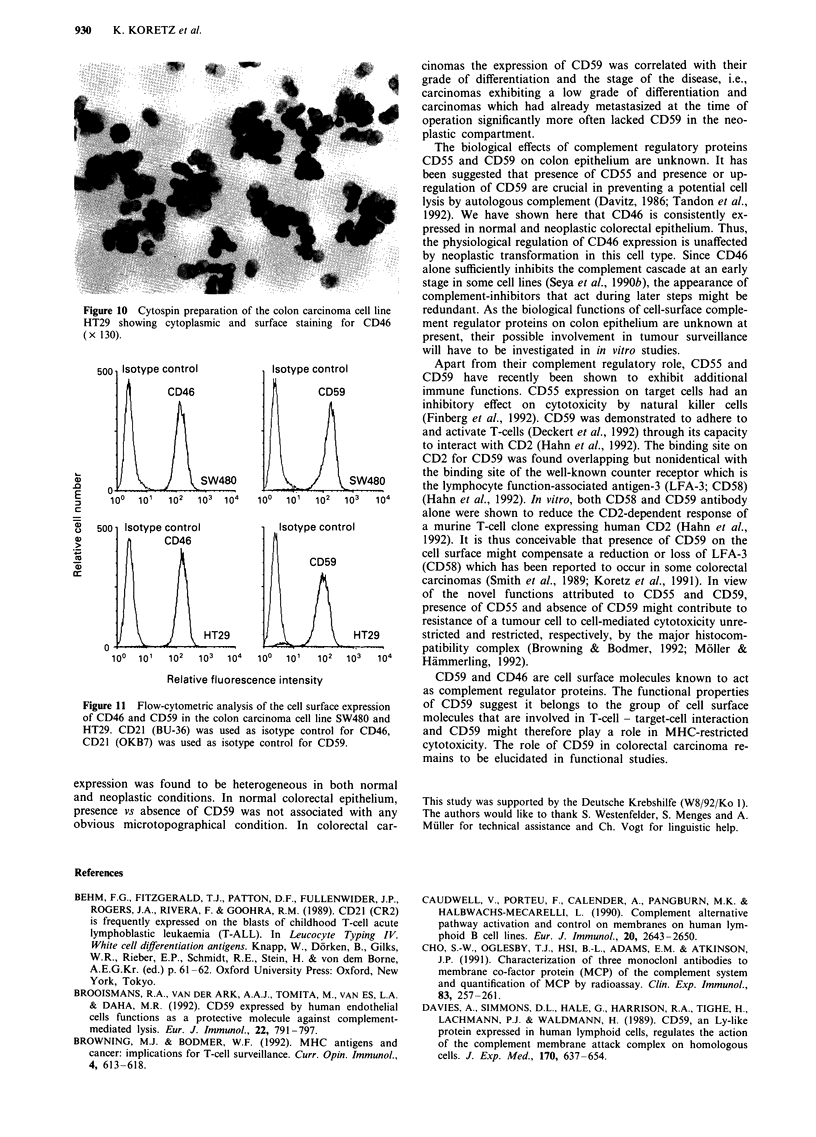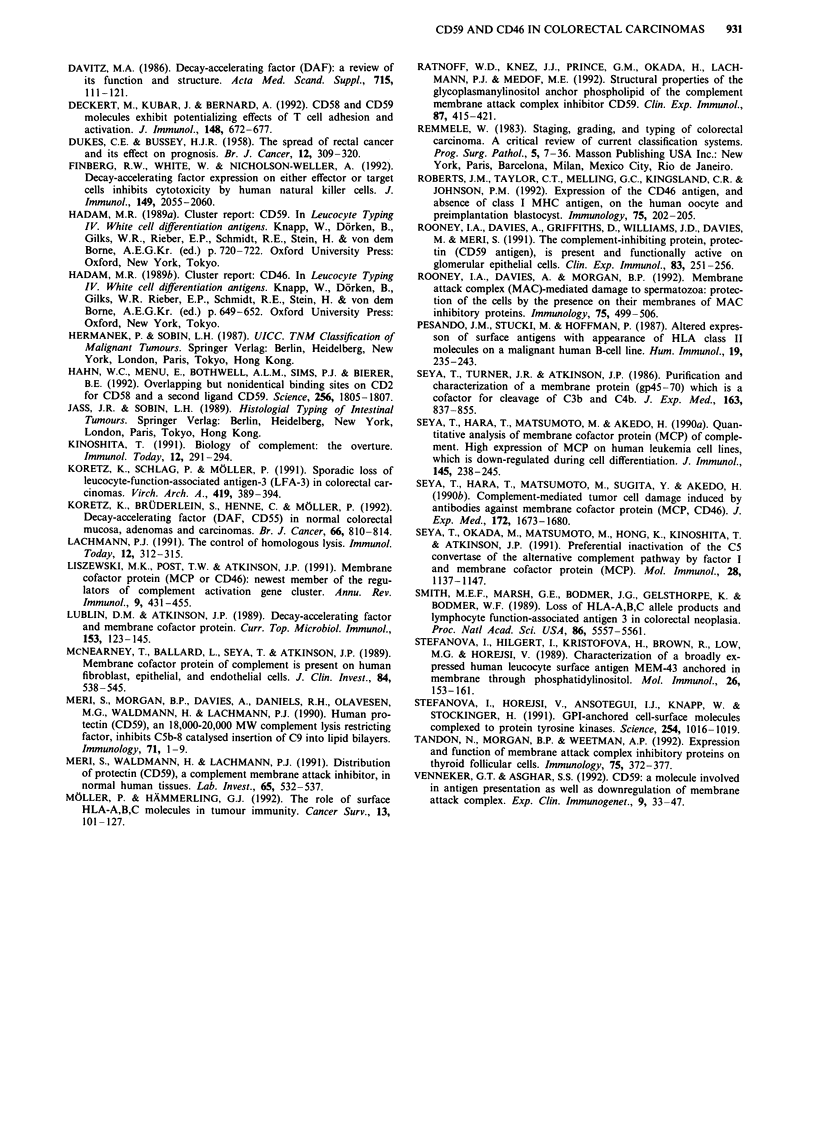# Expression of CD59, a complement regulator protein and a second ligand of the CD2 molecule, and CD46 in normal and neoplastic colorectal epithelium.

**DOI:** 10.1038/bjc.1993.456

**Published:** 1993-11

**Authors:** K. Koretz, S. Brüderlein, C. Henne, P. Möller

**Affiliations:** Institute of Pathology, University of Heidelberg, Germany.

## Abstract

**Images:**


 © Macmillan Press Ltd., 1993

### Expression of CD59, a complement regulator protein and a second ligand of the CD2 molecule, and CD46 in normal and neoplastic colorectal epithelium

K. Koretz, S. Brüderlein, C. Henne & P. Möller

*Institute of Pathology, University of Heidelberg, 69120 Heidelberg, Germany.*

**Summary** CD59 (protectin) and CD46 (membrane cofactor protein, MCP) are membrane-bound complement regulator proteins which inhibit complement-mediated cytolysis of autologous cells. CD59, a phosphatidyl-inositol-anchored glycoprotein, inhibits the formation of the terminal membrane attack complex (MAC) of complement and was found to be a second ligand for CD2 contributing to T-cell activation. In 20 colorectal normal mucosa samples, in ten adenomas, 71 carcinomas and in ten liver metastases derived thereof, CD59 was inconsistently expressed in the epithelial compartment. In carcinomas CD59 expression in the whole neoplastic compartment was more often found in well- and moderately differentiated tumours. By contrast, focal expression or even complete lack of CD59 was more often found in poorly differentiated tumours ($P = 0.021$). In addition, carcinomas without metastases at the time of operation (Dukes A/B) more often expressed CD59 in the entire neoplastic population compared to those carcinomas which had already metastasised ($P = 0.018$). There was no correlation between the mode of CD59 expression in colorectal carcinomas and the tumour type or location. CD46 has C3b/C4b binding and factor-I dependent cofactor activity and is broadly expressed in various cells and tissues. In the epithelial compartment of normal colorectal mucosa, of all adenomas, carcinomas and their liver metastases, CD46 was expressed throughout the epithelial compartment.

Since CD46 was consistently expressed in colorectal carcinomas the low expression or even lack of CD59 in a subset of tumours might not lead to critical complement-mediated attack of CD59-negative tumour cells. Regarding CD59 as a natural T-cell ligand involved in cognate T-cell – target-cell interaction, however, loss of CD59 might well be a selection advantage, provided that tumour antigen-mediated T-cell toxicity in colorectal carcinoma exists.

Membrane-bound regulatory proteins protect autologous cells from complement mediated cytotoxicity when fragments of the complement cascade are deposited on host cells which are not the desired target (Davitz, 1986). These proteins regulate the coordinating points in the classic and alternative pathways, the formation of the C3 convertases and the assembly of the terminal membrane attack complex (MAC) (Kinoshita, 1991). One of these proteins which aids in regulating the latter is protectin or CD59 (Hadam, 1989*a*), an 18–20 kD phosphatidyl-inositol anchored glycoprotein (Meri *et al.*, 1990; Ratnoff *et al.*, 1992). CD59 restricts homologous lysis by binding to the C8 and C9 molecules during the MAC formation, thus disturbing the C8:C9 ratio in the MAC complex (Meri *et al.*, 1990; Lachmann, 1991). CD59 is broadly expressed in human tissues, in human blood cells and neoplastic haematopoietic cell lines (Davies *et al.*, 1989), vascular endothelia (Brooismans *et al.*, 1992), various ductal epithelia, in kidney, lung, skin and placenta (Lachmann, 1991; Meri *et al.*, 1991; Rooney *et al.*, 1991), in thyroid follicular cells (Tandon *et al.*, 1992), and spermatozoa (Rooney *et al.*, 1992). However, CD59 might be involved in other immune regulatory mechanisms, such as signal transduction in cells (Stefanova *et al.*, 1991) and T-cell activation and adhesion (Deckert *et al.*, 1992; Venneker & Asghar, 1992) where it functions as a second ligand for CD2 (Hahn *et al.*, 1992). One protein which helps regulate the formation of the C3 convertases on autologous cells (Kinoshita, 1991) and the C5 convertase of the alternative complement pathway (Seya *et al.*, 1991) is the 45–70 kD membrane cofactor protein (MCP) (Lublin & Atkinson, 1989) or CD46 (Hadam, 1989*b*). This is a glycoprotein which acts on C3b and C4b and, as a cofactor, induces their factor I-mediated degradation (Seya *et al.*, 1986; Liszewsky *et al.*, 1991). MCP is also broadly expressed, in human fibroblasts, epithelial, and endothelial cells (McNearney *et al.*, 1989), on human oocytes and the preimplantation blastocyst (Roberts *et al.*, 1992), on peripheral blood cells (Seya *et al.*, 1990*a*; Cho *et al.*, 1991), in neoplastic haematopoietic cell lines (Caudwell *et al.*, 1990; Seya *et al.*, 1990*a*; Cho *et al.*, 1991), and malignant epithelial cell lines.

Although membrane-bound complement regulatory proteins have been extensively investigated on haematopoietic and non-haematopoietic cell lines, *in situ* studies, especially of solid tissues and their tumours, are still sparse. Here we present data on the expression of CD59 and CD46 in the normal colonic mucosa, in colorectal adenomas and carcinomas, and in liver metastases derived there from. To assess a possible prognostic potential the data were correlated with a set of well-established tumour parameters.

### Materials and methods

### *Immunohisto- and immunocytochemistry*

*Tissues and cells* Tissue samples from patients who underwent tumour resection of the colon or rectum reached our laboratory within 1 h after removal. These samples were obtained from cancers, from unaffected mucosa and from adenomas found in the removed specimens. They were quick-frozen in liquid nitrogen and stored at – 70°C until sectioning. Serial sections of 4 to 6 μm thickness were cut, thoroughly air dried, fixed in acetone for 10 min at room temperature, then stained immediately or stored at – 20°C for a short time. The collection comprised 20 tissue samples of unaffected mucosa, ten adenomas, 71 carcinomas and ten associated liver metastases. The tumours, whose primary site and metastatic spread at the time of operation were well documented, were typed, graded, and staged according to the International Union Against Cancer (UICC) classification (modified staging of the Dukes'-scheme (Dukes & Bussey, 1958) according to Turnbull *et al.* (Remmele, 1983); Hermanek & Sobin, 1987; Jass & Sobin, 1989). There were six carcinomas classified as grade I, 46 grade II, and 19 as grade III; 57 were non-mucinous and 14 were mucinous adenocar-

Correspondence: K. Koretz, Institute of Pathology, University of Heidelberg, Im Neuenheimer Feld 220, 69120 Heidelberg, Germany.
Received 12 January 1993; and in revised form 2 June 1993.

cinomas. According to the modified Dukes' staging there were 15 stage-A patients, 26 stage-B pateints, 20 stage-C patients and ten stage-D patients. Seventeen carcinomas were located at the right side of the colon and 54 at the left side. The colon carcinoma cell lines HT29 and SW480 (ATCC, Rockville, Maryland, USA) were raised in RPMI 1640 medium (Gibco, Paisley, Scotland, UK) which contained 10% foetal calf serum, sodium pyruvate and L-glutamine. The cells were detached with 0.25% ethylene-diamine-tetraacetate (EDTA), centrifuged at 1,000 r.p.m. for 5 min, and washed in RPMI 1640. Cytospin preparations were made, air-dried, fixed in acetone for 10 min, and stained immediately or stored at − 20°C.

*Reagents and staining procedure* CD59 (MEM-43( Stefanova *et al.*, 1989; IgG2a isotype; Serva, Heidelberg, Germany) and CD46 (J4–48) (Pesando *et al.*, 1987; IgG1 isotype; Dianova-Immunotech, Hamburg, Germany) were used for immunohistochemical detection of CD59 and CD46 antigen, respectively (Hadam, 1989*a*,*b*). Monoclonal antibody binding was detected with a polyclonal biotinylated sheep antibody to mouse immunoglobulin (Amersham, High Wycombe, UK) and a streptavidin-biotinylated peroxidase complex (Amersham). 3-Amino-9-ethylcarbazole (AEC) and N'N'-dimethylformamide (DMF) were obtained from Sigma Chemical Co. (St. Louis, Missouri, USA). Original preparations of CD59 and CD46 monoclonal antibody were diluted in the ratios 1:2,000 and 1:50 in PBS, respectively; the original preparation of biotinylated sheep antiserum to mouse immunoglobulin was diluted 1:50 in PBS, and the streptavidin peroxidase complex was diluted 1:100. Incubation times were 1 h at room temperature for the primary antibodies and 30 min for the second and third step reagents. Using AEC as the chromogen (0.4 mg ml$^{-1}$ in 0.01% $H_2O_2$ for 30 min), the peroxidase reaction caused a bright red precipitate. The sections were rinsed in tapwater, counterstained in Harris' hematoxylin and mounted with glycerol gelatin. Isotype-matched controls with irrelevant mAb, carried out on a limited number of normal mucosae and colon carcinomas, revealed no isotype-associated side reactions in or on epithelial cells. Each frozen section series contained a negative control without the primary reagent. In this, staining was observed solely in granulocytes whose endogeneous peroxidase was not blocked to achieve optimal antigenicity; though to a much lesser extent, staining was also observed in some epithelial areas, where it was assumed to be due to endogeneous biotin.

*Evaluation* A semiquantitative evaluation system was used to determine the antigen expression in normal, adenoma, and carcinoma tissue and cells by two of us (K.K. and P.M.). Antigen expression was scored '+' whenever specific staining was detectable, and '−' when no antigen was detectable. To give an indication of the relative numbers of stained and unstained cells, sections were scored '+ > −' when stained cells clearly outnumbered the unstained cells; '+/−' when positive and negative cells were found in equal proportions; '− > +' when unstained cells outnumbered the stained cells. Using this sytem, the antigen expression was correlated with the tumour grade, type, Dukes' stage and location of the tumour along the large bowel. For statistical analysis Fisher's exact test was used. The intensity of antigen expression was verbally described, if necessary. Stromal structures that were broadly positive for both CD59 and CD46, were used as internal standard to evaluate normal and neoplastically transformed epithelium.

### Flow cytometry

For flow cytometry 1 × 10$^6$ cells of HT29 and SW480 were used per sample. Cells were suspended in RPMI 1640 supplemented with 2% FCS, 10 mM HEPES and 0.1% $NaN_3$, referred to as FACS-medium. CD21 (OKB7) was used as isotype control for CD59 (IgG2a), CD21 (BU-36) was used as isotype control for CD46 (IgG1) (Behm *et al.*, 1989). The cells were incubated with the CD59 and CD46 antibodies, diluted 1:200 and 1:10, respectively, and, after three washing steps, with a polyclonal FITC-coupled F(ab')$_2$ goat-anti-mouse IgG + IgM (Dianova-Immunotech, Hamburg, Germany), diluted 1:50. The incubation time for each antibody was 1 h. After three washing steps propidium iodide (1 µg ml$^{-1}$ diluted in FACS-medium) was used to exclude nonviable cells. Flow cytometry was performed on a FACScan (Becton Dickinson, Mountain View, California, USA) with the LYSIS II software programme.

### Results

### Immunohistochemistry

*CD59* In normal colorectal mucosa CD59 was inconsistently expressed in the epithelium. 7/20 cases expressed CD59, predominantly on the adluminal cell surface of the upper part of the crypts. In some cases the antigen intensity was weak. Some cases showed low amounts of CD59 antigen in the cytoplasm and at the lateral cell borders (Figure 1). 3/20 mucosae expressed CD59 only in about half of the epithelial compartments; 10 samples entirely lacked CD59 (Table I). Despite this heterogeneous expression of CD59 in the epithelium, CD59 was broadly expressed in the sessile and mobile cells of the gut wall. The endothelial cells of small and large blood vessels strongly expressed CD59, as did the nerve fibre bundles of the autonomic plexus. Furthermore, fibrillar structures and fibroblasts in the interstitium were CD59 positive. The smooth muscle of the gut wall and in the arterial vessels expressed CD59 barely at all. Mononuclear cells of the mucosa and lymphoid cells in lymph follicles and in outer layers of the gut wall strongly expressed CD59.

Seven out of ten colorectal adenomas showed CD59 expression which was concentrated on the apical cell surface of the adenoma cells (Figure 2). One adenoma lacked CD59 in about half of the epithelial cells and two others lacked CD59 entirely (Table I).

In colorectal carcinomas CD59 expression was also heterogeneous. 42/71 cases showed CD59 antigen in all tumour cells, concentrated again on the apical cell border. 7/71 had more positive than negative neoplastic cells, 6/71 showed CD59 positive and negative tumour cells in about equal parts; 5/71 tumours were predominantly negative and 11/71 entirely lacked CD59 in their epithelial compartment (Figure 3). Stromal cells showed the same staining pattern as the normal gut wall. In addition, fibrillar structures and fibroblasts of the tumour stroma strongly expressed CD59.

**Figure 1** Expression of CD59 in normal colon mucosa. Prominent expression of CD59 in the upper part of the crypts. The CD59 antigen is concentrated at the luminal cell surface. Stromal cells of the mucosa express a high level of CD59 scale bar = 77 µm.

**Table I** Expression of CD59 and CD46 in the epithelial compartment of normal colorectal mucosa, adenomas and carcinomas

| Score | *Normal mucosa n = 20* CD59 | CD46 | *Adenomas n = 10* CD59 | CD46 | *Carcinomas n = 71* CD59 | CD46 |
|---|---|---|---|---|---|---|
| + | 7 | 20 | 7 | 10 | 42 (59.2%) | 71 |
| + > − | 0 | 0 | 0 | 0 | 7 (9.8%) | 0 |
| +/− | 3 | 0 | 1 | 0 | 6 (8.5%) | 0 |
| − > + | 0 | 0 | 0 | 0 | 5 (7.0%) | 0 |
| − | 10 | 0 | 2 | 0 | 11 (15.5%) | 0 |

Notes: '+', weak or strong staining of CD59 and CD46 in all epithelial cells. '+ > −', more positive than negative cells. '+/−', positive and negative cells in about equal numbers. '− > +', more negative than positive cells. '−', no staining of CD59 and CD46.

**Figure 2** CD59 in a colon adenoma. Antigen density peaks at the luminal cell surface scale bar = 77 μm.

**Figure 4** The liver metastasis of a colon carcinoma is CD59-positive. The tumour border is marked by arrow heads. Sinusoidal cells are positive (arrow); hepatocytes are CD59-negative scale bar = 77 μm.

**Figure 3** Lack of CD59 in a colon carcinoma. The tumour nodules are completely negative, the few dark stained cells in the tumour nodules are lymphoid cells (some marked by arrows). The tumour stroma is strongly CD59-positive scale bar = 77 μm.

From 41 carcinomas of the Dukes A and B group, 29 expressed CD59 in the whole tumour and 12 showed a focal expression or a complete loss of CD59. In contrast, from 30 carcinomas of the Dukes C and D group, only 13 carcinomas expressed CD59 in the whole tumour compartment, whereas 17 carcinomas showed a focal expression or a complete loss. Tumours expressing CD59 in the whole epithelium were found more often in the Dukes A/B group than in Dukes C/D ($P = 0.018$), i.e. CD59 was more often expressed in tumours without lymph node or liver metastases than in tumours that had metastasised at the time of operation.

From 52 grade I and II carcinomas 35 expressed CD59 in the whole tumour and 17 showed a focal expression or a complete loss. In contrast, from 19 grade III carcinomas only seven expressed CD59 in the whole tumour and 12 showed a focal expression or a complete loss. Well- and moderately differentiated tumours more often expressed CD59 in the whole epithelial compartment compared to less differentiated tumours ($P = 0.021$).

There was no significant correlation between the mode of CD59 expression and the tumour type or location.

In the ten liver metastases CD59 expression mainly corresponded to that found in their primaries. In 6/10 metastases a similar expression of CD59 was found when compared with their primary tumours, in 1/10 a lower antigen expression was found, and in 3/10 a higher expression of CD59 was found in the metastases. Peritumorous hepatocytes of the liver metastases were CD59-negtive. The bile ducts and bile ductuli were strongly CD59-positive, as were the sinusoids and the endothelial cells of larger blood vessels (Figure 4).

*CD46* In normal colorectal mucosa CD46 was expressed throughout the epithelium with no microtopographic differences along the crypts. Although the cell membranes were prominently stained, the cytoplasmic compartment was strongly positive, too (Figure 5). CD46 was also broadly expressed in the sessile and mobile cells of the gut wall: CD46 antigen density was high in endothelial cells of small and large blood vessels, intermediate in smooth muscle cells of the gut wall and the blood vessels, as well as in the nerve fibre bundles of the gut plexus, and low in a subset of fibroblasts and fibrillar structures. Most of the mononuclear cells within the lamina propria were CD46-positive. Lymphocytic cells and dendritic cells of lymph follicles strongly expressed CD46.

**Figure 5** Expression of CD46 in normal colon mucosa. Colon epithelium and stromal cells are strongly CD46-positive scale bar = 77 μm.

**Figure 6** CD46 in a colon adenoma. The antigen is confined to the cell membrane and, in lower antigen density, to the cytoplasm of the tumour cells scale bar = 77 μm.

**Figure 7** CD46 in a moderately differentiated non-mucinous colon adenocarcinoma. Neoplastic epithelium is strongly CD46-positive whereas the peritumoral stroma is only slightly positive scale bar = 77 μm.

**Figure 8** CD46 expression in colon carcinoma cells of a liver metastasis. The antigen is predominantly located at the cell surface scale bar = 77 μm.

In colorectal adenomas CD46 was found in the epithelium of all ten tumours with a staining pattern and antigen intensity corresponding to the CD46 expression in normal mucosa (Figure 6).

CD46 was consistently expressed in all colorectal carcinomas (Figure 7, Table I). The CD46 antigen density was often found to be higher in the neoplastic than in the adjacent non-neoplastic epithelium. The tumour stroma, i.e. fibroblasts and fibrillar structures, were slightly CD46-positive.

The cells of the liver metastases examined were likewise strongly CD46-positive (Figure 8). CD46 expression was at a high level in the biliary ductuli and ducts and in the sinusoidal system of the surrounding liver parenchyma, the hepatocytes were slightly CD46-positive.

*Immunocytochemistry and FACScan® analysis*

Cytospin preparations from SW480 and HT29 showed a strong expression of CD59 (Figure 9) and CD46 (Figure 10) in the cytoplasm. The maximum antigen density, however, was confined to the cell membrane. The FACScan® analysis demonstrated that under cell culture conditions both complement regulatory proteins were strongly surface-expressed on both colon carcinoma lines (Figure 11).

**Figure 9** Cytospin preparation of the colon carcinoma cell line SW480 showing CD59 in the cytoplasm and on the cell membrane (× 130).

### Discussion

Recently, we have reported that the decay-accelerating factor (DAF, CD55) is only sporadically expressed in colorectal epithelium, but is up-regulated in considerable percentages of colon adenomas and carcinomas. Expression was especially high in carcinomas of the mucinous type (Koretz *et al.*, 1992), which itself has a poorer prognosis than non-mucinous colorectal carcinomas. The present study adds further insight in the expression of complement regulatory membrane proteins in normal and neoplastic colorectal epithelium. CD59

**Figure 10** Cytospin preparation of the colon carcinoma cell line HT29 showing cytoplasmic and surface staining for CD46 (× 130).

**Figure 11** Flow-cytometric analysis of the cell surface expression of CD46 and CD59 in the colon carcinoma cell line SW480 and HT29. CD21 (BU-36) was used as isotype control for CD46, CD21 (OKB7) was used as isotype control for CD59.

expression was found to be heterogeneous in both normal and neoplastic conditions. In normal colorectal epithelium, presence *vs* absence of CD59 was not associated with any obvious microtopographical condition. In colorectal carcinomas the expression of CD59 was correlated with their grade of differentiation and the stage of the disease, i.e., carcinomas exhibiting a low grade of differentiation and carcinomas which had already metastasized at the time of operation significantly more often lacked CD59 in the neoplastic compartment.

The biological effects of complement regulatory proteins CD55 and CD59 on colon epithelium are unknown. It has been suggested that presence of CD55 and presence or upregulation of CD59 are crucial in preventing a potential cell lysis by autologous complement (Davitz, 1986; Tandon *et al.*, 1992). We have shown here that CD46 is consistently expressed in normal and neoplastic colorectal epithelium. Thus, the physiological regulation of CD46 expression is unaffected by neoplastic transformation in this cell type. Since CD46 alone sufficiently inhibits the complement cascade at an early stage in some cell lines (Seya *et al.*, 1990*b*), the appearance of complement-inhibitors that act during later steps might be redundant. As the biological functions of cell-surface complement regulator proteins on colon epithelium are unknown at present, their possible involvement in tumour surveillance will have to be investigated in *in vitro* studies.

Apart from their complement regulatory role, CD55 and CD59 have recently been shown to exhibit additional immune functions. CD55 expression on target cells had an inhibitory effect on cytotoxicity by natural killer cells (Finberg *et al.*, 1992). CD59 was demonstrated to adhere to and activate T-cells (Deckert *et al.*, 1992) through its capacity to interact with CD2 (Hahn *et al.*, 1992). The binding site on CD2 for CD59 was found overlapping but nonidentical with the binding site of the well-known counter receptor which is the lymphocyte function-associated antigen-3 (LFA-3; CD58) (Hahn *et al.*, 1992). *In vitro*, both CD58 and CD59 antibody alone were shown to reduce the CD2-dependent response of a murine T-cell clone expressing human CD2 (Hahn *et al.*, 1992). It is thus conceivable that presence of CD59 on the cell surface might compensate a reduction or loss of LFA-3 (CD58) which has been reported to occur in some colorectal carcinomas (Smith *et al.*, 1989; Koretz *et al.*, 1991). In view of the novel functions attributed to CD55 and CD59, presence of CD55 and absence of CD59 might contribute to resistance of a tumour cell to cell-mediated cytotoxicity unrestricted and restricted, respectively, by the major histocompatibility complex (Browning & Bodmer, 1992; Möller & Hämmerling, 1992).

CD59 and CD46 are cell surface molecules known to act as complement regulator proteins. The functional properties of CD59 suggest it belongs to the group of cell surface molecules that are involved in T-cell – target-cell interaction and CD59 might therefore play a role in MHC-restricted cytotoxicity. The role of CD59 in colorectal carcinoma remains to be elucidated in functional studies.

This study was supported by the Deutsche Krebshilfe (W8/92/Ko 1). The authors would like to thank S. Westenfelder, S. Menges and A. Müller for technical assistance and Ch. Vogt for linguistic help.

### References

BEHM, F.G., FITZGERALD, T.J., PATTON, D.F., FULLENWIDER, J.P., ROGERS, J.A., RIVERA, F. & GOOHRA, R.M. (1989). CD21 (CR2) is frequently expressed on the blasts of childhood T-cell acute lymphoblastic leukaemia (T-ALL). In *Leucocyte Typing IV. White cell differentiation antigens*. Knapp, W., Dörken, B., Gilks, W.R., Rieber, E.P., Schmidt, R.E., Stein, H. & von dem Borne, A.E.G.Kr. (ed.) p. 61–62. Oxford University Press: Oxford, New York, Tokyo.

BROOISMANS, R.A., VAN DER ARK, A.A.J., TOMITA, M., VAN ES, L.A. & DAHA, M.R. (1992). CD59 expressed by human endothelial cells functions as a protective molecule against complement-mediated lysis. *Eur. J. Immunol.*, **22,** 791–797.

BROWNING, M.J. & BODMER, W.F. (1992). MHC antigens and cancer: implications for T-cell surveillance. *Curr. Opin. Immunol.*, **4,** 613–618.

CAUDWELL, V., PORTEU, F., CALENDER, A., PANGBURN, M.K. & HALBWACHS-MECARELLI, L. (1990). Complement alternative pathway activation and control on membranes on human lymphoid B cell lines. *Eur. J. Immunol.*, **20,** 2643–2650.

CHO, S.-W., OGLESBY, T.J., HSI, B.-L., ADAMS, E.M. & ATKINSON, J.P. (1991). Characterization of three monoclonl antibodies to membrane co-factor protein (MCP) of the complement system and quantification of MCP by radioassay. *Clin. Exp. Immunol.*, **83,** 257–261.

DAVIES, A., SIMMONS, D.L., HALE, G., HARRISON, R.A., TIGHE, H., LACHMANN, P.J. & WALDMANN, H. (1989). CD59, an Ly-like protein expressed in human lymphoid cells, regulates the action of the complement membrane attack complex on homologous cells. *J. Exp. Med.*, **170,** 637–654.

DAVITZ, M.A. (1986). Decay-accelerating factor (DAF): a review of its function and structure. *Acta Med. Scand. Suppl.*, **715**, 111-121.

DECKERT, M., KUBAR, J. & BERNARD, A. (1992). CD58 and CD59 molecules exhibit potentializing effects of T cell adhesion and activation. *J. Immunol.*, **148**, 672-677.

DUKES, C.E. & BUSSEY, H.J.R. (1958). The spread of rectal cancer and its effect on prognosis. *Br. J. Cancer*, **12**, 309-320.

FINBERG, R.W., WHITE, W. & NICHOLSON-WELLER, A. (1992). Decay-accelerating factor expression on either effector or target cells inhibits cytotoxicity by human natural killer cells. *J. Immunol.*, **149**, 2055-2060.

HADAM, M.R. (1989*a*). Cluster report: CD59. In *Leucocyte Typing IV. White cell differentiation antigens*. Knapp, W., Dörken, B., Gilks, W.R., Rieber, E.P., Schmidt, R.E., Stein, H. & von dem Borne, A.E.G.Kr. (ed.) p. 720-722. Oxford University Press: Oxford, New York, Tokyo.

HADAM, M.R. (1989*b*). Cluster report: CD46. In *Leucocyte Typing IV. White cell differentiation antigens*. Knapp, W., Dörken, B., Gilks, W.R. Rieber, E.P., Schmidt, R.E., Stein, H. & von dem Borne, A.E.G.Kr. (ed.) p. 649-652. Oxford University Press: Oxford, New York, Tokyo.

HERMANEK, P. & SOBIN, L.H. (1987). *UICC. TNM Classification of Malignant Tumours*. Springer Verlag: Berlin, Heidelberg, New York, London, Paris, Tokyo, Hong Kong.

HAHN, W.C., MENU, E., BOTHWELL, A.L.M., SIMS, P.J. & BIERER, B.E. (1992). Overlapping but nonidentical binding sites on CD2 for CD58 and a second ligand CD59. *Science*, **256**, 1805-1807.

JASS, J.R. & SOBIN, L.H. (1989). *Histologial Typing of Intestinal Tumours*. Springer Verlag: Berlin, Heidelberg, New York, London, Paris, Tokyo, Hong Kong.

KINOSHITA, T. (1991). Biology of complement: the overture. *Immunol. Today*, **12**, 291-294.

KORETZ, K., SCHLAG, P. & MÖLLER, P. (1991). Sporadic loss of leucocyte-function-associated antigen-3 (LFA-3) in colorectal carcinomas. *Virch. Arch. A.*, **419**, 389-394.

KORETZ, K., BRÜDERLEIN, S., HENNE, C. & MÖLLER, P. (1992). Decay-accelerating factor (DAF, CD55) in normal colorectal mucosa, adenomas and carcinomas. *Br. J. Cancer*, **66**, 810-814.

LACHMANN, P.J. (1991). The control of homologous lysis. *Immunol. Today*, **12**, 312-315.

LISZEWSKI, M.K., POST, T.W. & ATKINSON, J.P. (1991). Membrane cofactor protein (MCP or CD46): newest member of the regulators of complement activation gene cluster. *Annu. Rev. Immunol.*, **9**, 431-455.

LUBLIN, D.M. & ATKINSON, J.P. (1989). Decay-accelerating factor and membrane cofactor protein. *Curr. Top. Microbiol. Immunol.*, **153**, 123-145.

MCNEARNEY, T., BALLARD, L., SEYA, T. & ATKINSON, J.P. (1989). Membrane cofactor protein of complement is present on human fibroblast, epithelial, and endothelial cells. *J. Clin. Invest.*, **84**, 538-545.

MERI, S., MORGAN, B.P., DAVIES, A., DANIELS, R.H., OLAVESEN, M.G., WALDMANN, H. & LACHMANN, P.J. (1990). Human protectin (CD59), an 18,000-20,000 MW complement lysis restricting factor, inhibits C5b-8 catalysed insertion of C9 into lipid bilayers. *Immunology*, **71**, 1-9.

MERI, S., WALDMANN, H. & LACHMANN, P.J. (1991). Distribution of protectin (CD59), a complement membrane attack inhibitor, in normal human tissues. *Lab. Invest.*, **65**, 532-537.

MÖLLER, P. & HÄMMERLING, G.J. (1992). The role of surface HLA-A,B,C molecules in tumour immunity. *Cancer Surv.*, **13**, 101-127.

RATNOFF, W.D., KNEZ, J.J., PRINCE, G.M., OKADA, H., LACHMANN, P.J. & MEDOF, M.E. (1992). Structural properties of the glycoplasmanylinositol anchor phospholipid of the complement membrane attack complex inhibitor CD59. *Clin. Exp. Immunol.*, **87**, 415-421.

REMMELE, W. (1983). Staging, grading, and typing of colorectal carcinoma. A critical review of current classification systems. *Prog. Surg. Pathol.*, **5**, 7-36. Masson Publishing USA Inc.: New York, Paris, Barcelona, Milan, Mexico City, Rio de Janeiro.

ROBERTS, J.M., TAYLOR, C.T., MELLING, G.C., KINGSLAND, C.R. & JOHNSON, P.M. (1992). Expression of the CD46 antigen, and absence of class I MHC antigen, on the human oocyte and preimplantation blastocyst. *Immunology*, **75**, 202-205.

ROONEY, I.A., DAVIES, A., GRIFFITHS, D., WILLIAMS, J.D., DAVIES, M. & MERI, S. (1991). The complement-inhibiting protein, protectin (CD59 antigen), is present and functionally active on glomerular epithelial cells. *Clin. Exp. Immunol.*, **83**, 251-256.

ROONEY, I.A., DAVIES, A. & MORGAN, B.P. (1992). Membrane attack complex (MAC)-mediated damage to spermatozoa: protection of the cells by the presence on their membranes of MAC inhibitory proteins. *Immunology*, **75**, 499-506.

PESANDO, J.M., STUCKI, M. & HOFFMAN, P. (1987). Altered expresson of surface antigens with appearance of HLA class II molecules on a malignant human B-cell line. *Hum. Immunol.*, **19**, 235-243.

SEYA, T., TURNER, J.R. & ATKINSON, J.P. (1986). Purification and characterization of a membrane protein (gp45-70) which is a cofactor for cleavage of C3b and C4b. *J. Exp. Med.*, **163**, 837-855.

SEYA, T., HARA, T., MATSUMOTO, M. & AKEDO, H. (1990*a*). Quantitative analysis of membrane cofactor protein (MCP) of complement. High expression of MCP on human leukemia cell lines, which is down-regulated during cell differentiation. *J. Immunol.*, **145**, 238-245.

SEYA, T., HARA, T., MATSUMOTO, M., SUGITA, Y. & AKEDO, H. (1990*b*). Complement-mediated tumor cell damage induced by antibodies against membrane cofactor protein (MCP, CD46). *J. Exp. Med.*, **172**, 1673-1680.

SEYA, T., OKADA, M., MATSUMOTO, M., HONG, K., KINOSHITA, T. & ATKINSON, J.P. (1991). Preferential inactivation of the C5 convertase of the alternative complement pathway by factor I and membrane cofactor protein (MCP). *Mol. Immunol.*, **28**, 1137-1147.

SMITH, M.E.F., MARSH, G.E., BODMER, J.G., GELSTHORPE, K. & BODMER, W.F. (1989). Loss of HLA-A,B,C allele products and lymphocyte function-associated antigen 3 in colorectal neoplasia. *Proc. Natl Acad. Sci. USA*, **86**, 5557-5561.

STEFANOVA, I., HILGERT, I., KRISTOFOVA, H., BROWN, R., LOW, M.G. & HOREJSI, V. (1989). Characterization of a broadly expressed human leucocyte surface antigen MEM-43 anchored in membrane through phosphatidylinositol. *Mol. Immunol.*, **26**, 153-161.

STEFANOVA, I., HOREJSI, V., ANSOTEGUI, I.J., KNAPP, W. & STOCKINGER, H. (1991). GPI-anchored cell-surface molecules complexed to protein tyrosine kinases. *Science*, **254**, 1016-1019.

TANDON, N., MORGAN, B.P. & WEETMAN, A.P. (1992). Expression and function of membrane attack complex inhibitory proteins on thyroid follicular cells. *Immunology*, **75**, 372-377.

VENNEKER, G.T. & ASGHAR, S.S. (1992). CD59: a molecule involved in antigen presentation as well as downregulation of membrane attack complex. *Exp. Clin. Immunogenet.*, **9**, 33-47.